# Critical reflection on the indication for computed tomography: an interdisciplinary survey of risk and benefit management in patients with sepsis

**DOI:** 10.1186/s13244-024-01894-3

**Published:** 2025-01-13

**Authors:** Maria Isabel Opper Hernando, Denis Witham, Ann-Christine Stahl, Peter Richard Steinhagen, Stefan Angermair, Wolfgang Bauer, Friederike Compton, Andreas Edel, Jan Matthias Kruse, York Kühnle, Gunnar Lachmann, Susanne Marz, Holger Müller-Redetzky, Jens Nee, Oliver Paul, Damaris Praeger, Carsten Skurk, Miriam Stegemann, Alexander Uhrig, Stefan Wolf, Myrto Bolanaki, Kerstin Rubarth, Joachim Seybold, Elke Zimmermann, Marc Dewey, Julian Pohlan

**Affiliations:** 1 Department of Radiology, Charité – Universitätsmedizin Berlin, Freie Universität Berlin and Humboldt- Universität zu Berlin, Charitéplatz 1, Berlin, Germany; 2 Department of Cardiology, Charité – Universitätsmedizin Berlin, Freie Universität Berlin and Humboldt- Universität zu Berlin, Charitéplatz 1, Berlin, Germany; 3 Berlin Institute of Health, Charité – Universitätsmedizin Berlin, Charitéplatz 1, Berlin, Germany; 4 Department of Gastroenterology and Hepatology, Charité – Universitätsmedizin Berlin, Freie Universität Berlin and Humboldt- Universität zu Berlin, Charitéplatz 1, Berlin, Germany; 5 Department of Anesthesiology and Intensive Care Medicine, Charité – Universitätsmedizin Berlin, Freie Universität Berlin and Humboldt- Universität zu Berlin, Hindenburgdamm 30, Berlin, Germany; 6 Emergency Department, Charité – Universitätsmedizin Berlin, Freie Universität Berlin and Humboldt- Universität zu Berlin, Hindenburgdamm 30, Berlin, Germany; 7 Medical Clinic with Focus on Nephrology and Internal Intensive Care Medicine, Charité – Universitätsmedizin Berlin, Freie Universität Berlin and Humboldt- Universität zu Berlin, Hindenburgdamm 30, Berlin, Germany; 8 Department of Anesthesiology and Intensive Care Medicine (CCM, CVK), Charité – Universitätsmedizin Berlin, Freie Universität Berlin and Humboldt- Universität zu Berlin, Charitéplatz 1 and Augustenburger Platz 1, Berlin, Germany; 9 Medical Clinic with Focus on Nephrology and Internal Intensive Care Medicine, Charité – Universitätsmedizin Berlin, Freie Universität Berlin and Humboldt- Universität zu Berlin, Augustenburger Platz 1, Berlin, Germany; 10 Department of Cardiology, Charité – Universitätsmedizin Berlin, Freie Universität Berlin and Humboldt- Universität zu Berlin, Augustenburger Platz 1, Berlin, Germany; 11 Interdisciplinary Anesthesiology and Surgical Intensive Care Unit, Charité – Universitätsmedizin Berlin, Freie Universität Berlin and Humboldt- Universität zu Berlin, Charitéplatz 1 and Augustenburger Platz 1, Berlin, Germany; 12 Department of Infectious Diseases and Respiratory Medicine, Charité – Universitätsmedizin Berlin, Freie Universität Berlin and Humboldt- Universität zu Berlin, Charitéplatz 1, Berlin, Germany; 13 Clinic for Cardiology, Angiology and Intensive Care Medicine, Charité – Universitätsmedizin Berlin, Freie Universität Berlin and Humboldt- Universität zu Berlin, Charitéplatz 1, Berlin, Germany; 14 Department of Cardiology, Charité – Universitätsmedizin Berlin, Freie Universität Berlin and Humboldt- Universität zu Berlin, Hindenburgdamm 30, Berlin, Germany; 15 Department of Infectious Diseases and Respiratory Medicine, Charité – Universitätsmedizin Berlin, Freie Universität Berlin and Humboldt- Universität zu Berlin, Augustenburger Platz 1, Berlin, Germany; 16 Department of Neurosurgery with Pediatric Neurosurgery Unit, Charité – Universitätsmedizin Berlin, Freie Universität Berlin and Humboldt- Universität zu Berlin, Charitéplatz 1, Berlin, Germany; 17 Emergency Department, Charité – Universitätsmedizin Berlin, Freie Universität Berlin and Humboldt- Universität zu Berlin, Charitéplatz 1 and Augustenburger Platz 1, Berlin, Germany; 18 Institute of Biometry and Clinical Epidemiology, Charité – Universitätsmedizin Berlin, Freie Universität Berlin and Humboldt- Universität zu Berlin, Charitéplatz 1, Berlin, Germany; 19 Medical Directorate, Charité – Universitätsmedizin Berlin, Freie Universität Berlin and Humboldt- Universität zu Berlin, Charitéplatz 1, Berlin, Germany; 20 Department of Radiology, Oberhavel Kliniken – Oberhavel Kliniken GmbH, Academic Teaching Hospital of the Charité - Universitätsmedizin Berlin, Hennigsdorf, Germany; 21 Johnson & Johnson Innovative Medicine, Janssen-Cilag GmbH, Johnson & Johnson Platz 1, Neuss, Germany

**Keywords:** Tomography (X-ray computed), Sepsis, Surveys and questionnaires, Clinical reasoning, Contrast media

## Abstract

**Objectives:**

To survey physicians’ views on the risks and benefits of computed tomography (CT) in the management of septic patients and indications for and contraindications to contrast media use in searching for septic foci.

**Methods:**

A web-based questionnaire was administered to physicians at a large European university medical center in January 2022. A total of 371 questionnaires met the inclusion criteria and were analyzed with physicians’ work experience, workplace, and medical specialty as independent variables. Chi-square tests were used for exploratory analysis.

**Results:**

While physicians with all levels of work experience were included, the largest group (35.0%, *n* = 130/371) had 3–7 years of experience. Most physicians agreed that the benefits of CT outweigh its potential adverse effects in septic patients (90.5%, *n* = 336/371). Responders saw the strongest indication for contrast media administration in septic patients for (1) CT examinations of the abdomen (92.7%, *n* = 333/359) and (2) combined CT examinations of the chest, abdomen, and pelvis (94.1%, *n* = 337/358). While radiologists were most likely to consider manifest hyperthyroidism an absolute contraindication to contrast media administration (43.8%, *n* = 14/32), most other groups of physicians opted for appropriate preparation before contrast media administration in this subset of septic patients.

**Conclusion:**

In this survey, most participating physicians considered CT an essential diagnostic modality to detect an infectious focus in septic patients. Whereas the risk of ionizing radiation was regarded as justifiable by most physicians, different specialties varied in their assessment of the risks of contrast media administration.

**Key Points:**

- Physicians recognize CT as a relevant imaging modality in the diagnostic management of patients with sepsis.
- There is an interdisciplinary consensus that the use of ionizing radiation is justified in septic patients.
- There is disagreement about indications for and contraindications to contrast media administration among physicians from different medical specialties.

**Graphical Abstract:**

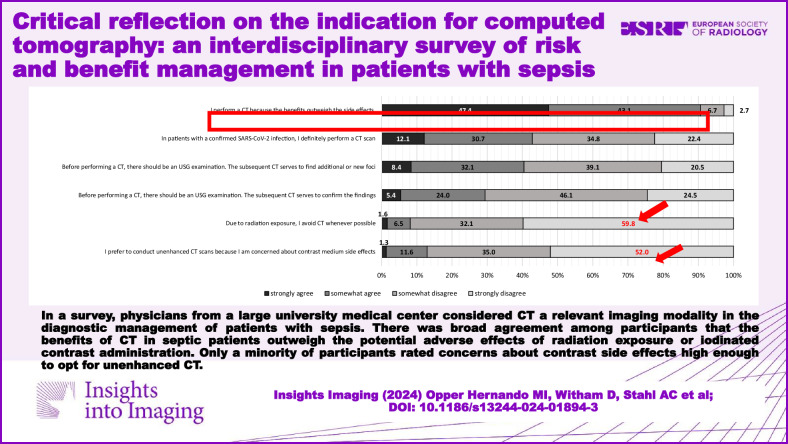

## Introduction

Sepsis is a life-threatening medical condition defined by a dysregulated host inflammatory response to infection that can culminate in multiple organ dysfunction syndrome [1, 2]. Time plays a key role in treating septic patients [3, 4]. Early recognition of sepsis is crucial to promptly initiate circulatory stabilization and antibiotic treatment, and so is the rapid and targeted control of the source of infection [1, 5, 6]. Rüddel et al found that each hour of delay in antimicrobial treatment increased mortality in septic patients with and without shock and heightened the risk of progression from sepsis to septic shock, while delayed surgical source control was specifically linked to increased mortality in patients with septic shock [4]. In addition to pinpointing potential infectious foci through the patient’s medical history and clinical evaluation, physicians can use diagnostic imaging modalities like ultrasonography (USG) and computed tomography (CT) to identify the source of sepsis [7–9]. An advantage of CT imaging is that it can provide detailed information on the condition of a patient’s organs and tissues in a short examination time [10, 11]. However, a CT scan has downsides due to exposure to ionizing radiation, which has the potential to increase a patient’s risk of cancer over time [12, 13]. Therefore, physicians must always check for the justification to perform a CT scan [14]. However, when considering alternative imaging procedures without ionizing radiation, one should not ignore the limiting factors of magnetic resonance imaging (MRI), such as the longer examination time compared to CT and its restricted availability [12].

A joint CT scan of the chest, abdomen, and pelvis (whole-body CT) is the most commonly conducted and preferred CT examination to detect infectious foci, such as pneumonia or abscesses, as these organ regions are the most common sites of septic foci [15–18]. To further improve diagnostic accuracy, it is common practice to perform CT scans with administration of iodinated contrast media (ICM). In patients with sepsis, contrast-enhanced CT (CECT) can be conducted to detect septic foci, to better assess the exact extent of the infection, and to initiate possible focal treatment such as abscess drainage. In daily clinical routine, many physicians weigh the diagnostic effectiveness of CECT against the possible risks such as (1) acute kidney injury (AKI) due to contrast-induced nephropathy (CIN), (2) mild adverse side effects up to severe anaphylaxis, and (3) iodinated contrast-induced thyrotoxicosis [19–21]. Therefore, individual assessment of each patient’s risk profile for complications after contrast administration is recommended [22]. Overall, sepsis remains a severe disease with high mortality worldwide, highlighting the need for evidence-based and clearly defined diagnostic and therapeutic protocols assisting physicians in the management of septic patients [1, 23]. This study aims to provide insight into physicians’ assessment of the risks and benefits of CT imaging and ICM administration in patients with sepsis.

## Methods

### Survey and setting

This cross-sectional study was approved by the staff council and the local ethics committee (EA1/203/21). The Declaration of Helsinki was respected. Consent was a prerequisite for participation. All physicians of a large German university medical center were contacted for possible participation in our prospective study. The questionnaire on the use of CT in patients with sepsis was developed by our interdisciplinary study team. We previously applied the survey in a cohort of final-year medical students [24]. Part of the results of our comprehensive analysis have been published separately [18].

### Questionnaire structure

**Fig. 1:**
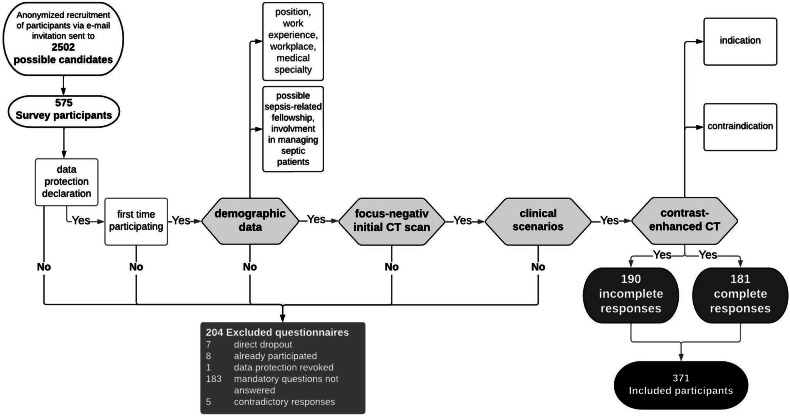
Flow chart of survey participation. A total of 2502 physicians were invited to participate in our survey, and 575 completed the questionnaire. Prerequisites for inclusion in the analysis were complete information on demographic data and complete answers to the question groups focus-negative initial CT scan and clinical scenarios. 204 questionnaires had to be excluded from analysis. Of the 371 questionnaires that met the inclusion criteria, 190 were incomplete in the contrast-enhanced CT category, while 181 participants answered all questions. Adapted from Fig. 1 of Opper Hernando et al [18]

Participants were asked to provide demographic data (Fig. 1). As the first part of the questionnaire has been previously published [18], the following questions pertain to the remaining data. The first subset of questions consisted of five options for how to proceed if no source of infection was detected on an initial CT scan. Participants were asked to rate their agreement with these options using a 4-point Likert scale: (1) strongly disagree, (2) somewhat disagree, (3) somewhat agree, and (4) strongly agree. In the second group of questions, physicians were presented with six clinical scenarios regarding the management of patients with sepsis to be answered using the same 4-point Likert scale. Complete answers to these two groups of questions were mandatory for inclusion in the study. In the third subset of questions, participants were asked about the indication for administration of ICM depending on the body region being examined by CT. Physicians could choose between the following options: (1) definitely without ICM, (2) preferably without ICM, (3) preferably with ICM, and (4) definitely with ICM. Finally, the fourth set of questions addressed the contraindications to ICM administration in septic patients and additionally one of the listed complications. The response options were: (1) absolute contraindication to ICM, (2) relative contraindication to ICM, (3) CT with ICM possible after appropriate preparation, and (4) no contraindication to ICM. The two sets of questions regarding ICM use were optional. Incomplete answers were no reason for exclusion.

### Administration and data handling

An email containing a link leading to the digital survey was sent to all (*n* = 2502) physicians of our university medical center using official distribution lists (Fig. 1). The link was active between the 3rd and the 31st of January, 2022. A total of 575 physicians (23.0% response rate) participated, of whom 64.5% (*n* = 371/575) met the inclusion criteria of completing the first two subsets of questions and participating for the first time.

### Data analysis

Data were collected anonymously through the online platform LimeSurvey (LimeSurvey Cloud, version 5.3.25, 2022; LimeSurvey GmbH, Hamburg, Germany) and extracted into Excel tables (Microsoft® Excel® for Microsoft 365 MSO, version 2112, 2017; Microsoft, Redmond, WA, USA). Demographics like work experience, workplace (> 50% of working time), and medical specialty were used as independent variables for statistical analysis. To avoid bias, one participant reporting two medical specialties was excluded from all analyses with this variable.

As inclusion criteria required at minimum the first two subsets of questions completed, the number of items analyzed may vary as reported throughout the manuscript. Descriptive statistics such as absolute and relative frequencies were gathered using SPSS (Statistical Package for the Social Sciences, IBM® SPSS Statistics, version 28.0.1.0, 2021; IBM, Armonk, NY, USA). More detailed descriptive data can be found in the supplementary material. Figures were designed using Excel. Statistical hypothesis test such as the chi-square test were performed by using SPSS for an exploratory interpretation of the data. A significance level of α < 0.05 was used for all analyses. However, due to the exploratory nature of the study, *p*-values are not adjusted for multiplicity and are interpreted in a hypothesis-generating manner. Acquired *p*-values are presented in Table 1.

**Table 1:** Compilation of *p*-values for effect of variables on responses (chi-square test)

		Work experience	Workplace	Medical specialty
		*p*-value	*p*-value	*p*-value
Which clinical scenario applies to you in the management of patients with sepsis?	I perform a CT scan for sepsis because the benefits outweigh adverse effects (e.g., radiation exposure, contrast media).	0.903	0.131	0.060
	In patients with a confirmed SARS-CoV-2 infection, I definitely perform a CT scan.	0.627	**0.006**	0.801
	Before I perform a CT scan, there should be an ultrasound examination. The subsequent CT serves to detect additional or new foci.	0.308	**0.015**	**0.001**
	Before I perform a CT scan, there should be an ultrasound examination. The subsequent CT serves to confirm the findings.	0.146	**0.002**	**0.001**
	Due to radiation exposure, I avoid CT in patients with sepsis whenever possible.	**0.028**	0.414	**0.005**
	I prefer to conduct unenhanced CT scans to avoid adverse effects of contrast media administration.	0.216	**0.008**	**0.000**
Indication for contrast-enhanced CT for focus search in individuals with sepsis.	Abdomen	**0.003**	**< 0.001**	**< 0.001**
	Chest	0.583	0.126	**0.019**
	Combined examination of the chest, abdomen, and pelvis	**0.014**	**< 0.001**	**< 0.001**
	Head (cranial CT)	0.372	0.331	**0.001**
	Neck	**0.038**	**< 0.001**	**< 0.001**
	Teeth	0.149	**0.001**	**< 0.001**
	Paranasal sinuses	0.146	**0.023**	**0.008**
	Legs	0.195	**< 0.001**	**< 0.001**
Contraindications to the use of contrast media for focus-CT in septic patients	Latent hyperthyroidism	0.387	0.131	0.080
	Manifest hyperthyroidism	0.391	**< 0.001**	**< 0.001**
	Creatinine > 2 mg/dL or eGFR < 30 mL/min	0.087	**< 0.001**	**0.003**
	End-stage renal failure/hemodialysis	**0.008**	**< 0.001**	**< 0.001**
	Slight acute adverse/allergic-type reaction	0.641	0.159	**0.002**
	Severe acute adverse/allergic-type reaction	0.515	0.102	0.431
	Acute heart failure/NYHA stage IV	**0.022**	0.074	0.074
	Clinical presentation with ileus	0.264	**< 0.001**	**0.009**
If the initial CT fails to detect a focus, …	… I would rely on further diagnostic tests	0.843	**0.007**	0.565
	… I request a re-CT in case of clinical deterioration	**0.031**	0.131	0.760
	… I would use alternative imaging modalities (e.g., USG, MRI, PET-CT)	0.105	**0.006**	**0.004**
	... and the patient is clinically unchanged, I request a re-CT scan after 3 days	0.169	**0.035**	**0.000**
	… and the patient has improved clinically, I would request a follow-up CT after 1 week	0.272	0.131	0.374

Three variables—work experience, workplace, and medical specialty—were tested for their effect on participants’ responses to each question. Significance was assumed for *p* < 0.05. Significant *p*-values are shown in bold. The supplementary material provides detailed insights into the descriptive analysis of some question groups

Latent hyperthyroidism: thyroid-stimulating hormone (TSH) reduced, free triiodothyronine (fT3) in normal range; manifest hyperthyroidism: TSH reduced, fT3 increased

*CT* computed tomography, *re-CT* repeat computed tomography, *USG* ultrasonography, *MRI* magnetic resonance imaging, *PET-CT* positron emission tomography and computed tomography, *SARS-CoV-2* severe acute respiratory syndrome coronavirus type 2, *eGFR* estimated glomerular filtration rate, *NYHA* New York Heart Association

## Results

### Study population

The majority of physicians participating in our survey had a work experience of > 3 to ≤ 7 years (35.0%, *n* = 130/371) (Table 2). One-third (31.0%, *n* = 115/370) of the participants worked in an intensive care unit (ICU). Radiologists made up the smallest and internal medicine physicians the largest proportion of the medical-specialties variable. Detailed demographic information can be found in Table 2 of our earlier publication entitled “Interdisciplinary perspectives on computed tomography in sepsis: survey among medical doctors at a large university medical center” [18] and Tables S1–S4 of the corresponding supplementary material.

**Table 2:** Demographic data of study participants included in analysis

		*n* = total number	Percentages (%)
Work experience *n* = 371	< 3 years	74	19.9
	> 3 to ≤ 7 years	130	35.0
	> 7 to ≤ 11 years	73	19.7
	> 11 to ≤ 20 years	64	17.3
	> 20 years	30	8.1
Medical specialty *n* = 370	Internal medicine	157	42.4
	Anesthesiology	70	18.9
	Other specialty	66	17.8
	Surgery	44	11.9
	Radiology	33	8.9
Workplace *n* = 371	Intensive care unit	115	31.0
	General ward	83	22.4
	Emergency department	49	13.2
	Operating room (OR)	42	11.3
	Radiology department	32	8.6
	Outpatient clinic	32	8.6
	Other	18	4.6
Do you deal with septic patients in your daily clinical practice? *n* = 371	Yes	346	93.3
	No	25	6.7

The demographic information has previously been published in Opper Hernando et al [18]. Portions are reiterated for clarity and comprehension

### How to manage patients with sepsis—CT-related clinical scenarios

**Fig. 2:**
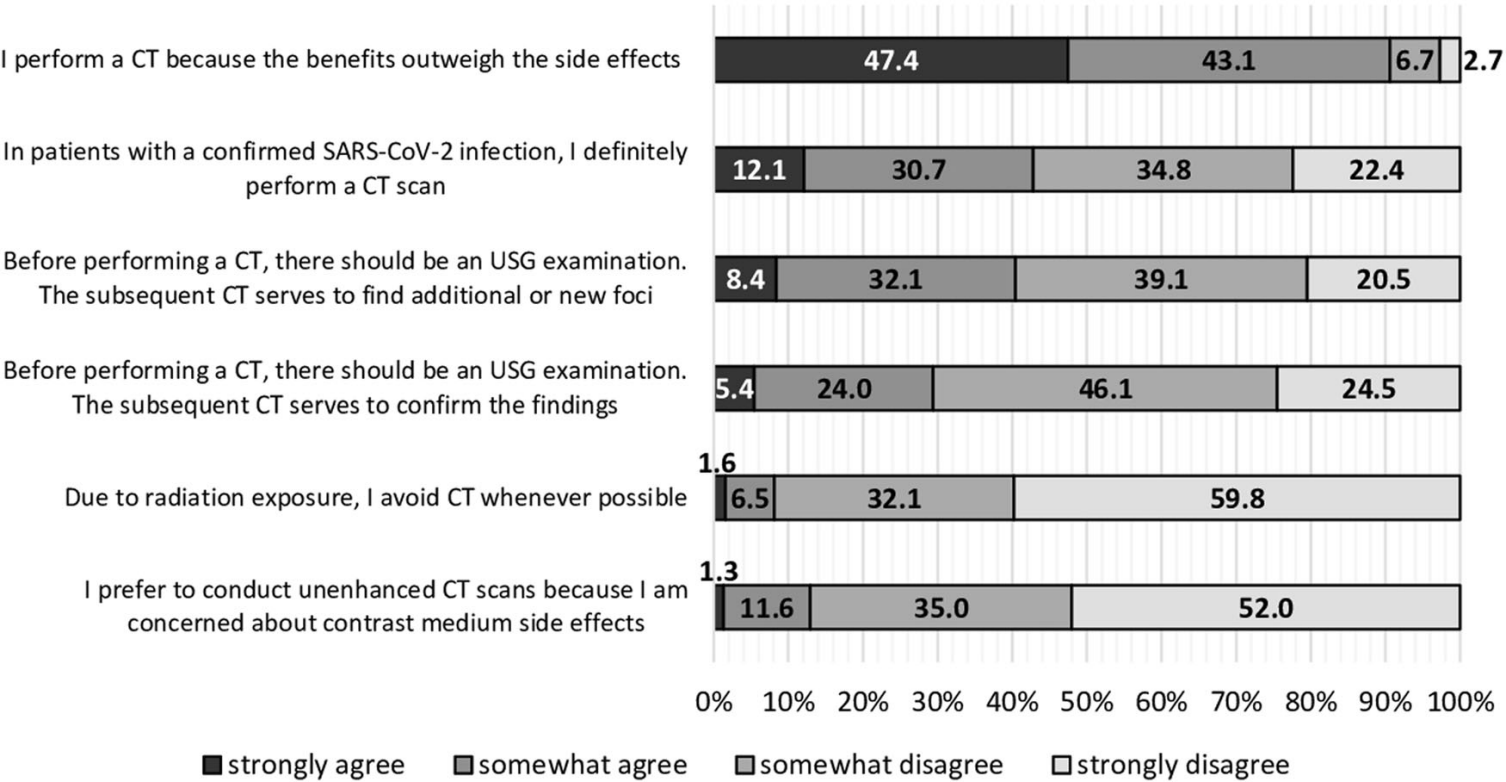
Clinical scenarios with regard to the conduct of CT examinations in the management of sepsis. 47.4% (*n* = 176/371) of participants strongly and 43.1% (*n* = 160/371) somewhat agreed that the benefits of a CT examination in septic patients outweigh potential adverse effects. Physicians most commonly disagreed to avoid a CT scan in patients with sepsis due to radiation exposure (91.9%, *n* = 341/371). 87.0% (*n* = 323/371) of the participants disagreed with the notion that unenhanced CT scans were preferable because of concerns about adverse effects of contrast media. Heterogeneous distributions of responses were also found for the statements “I perform a CT in patients with confirmed SARS-CoV-2 infection” and “the CT scan after an USG examination serves to detect additional and new foci.” Physicians mostly somewhat disagreed (34.8%, *n* = 129/371; 39.1%, *n* = 145/371; respectively) or somewhat agreed (30.7%, *n* = 114/371; 32.1%, *n* = 119/371) with these statements. Even more physicians disagreed that a USG examination should be performed before a CT examination, in order for the CT to confirm the USG findings (70.6%, *n* = 262/371). For more detailed descriptive information, see the supplementary material (Tables S1–S3). CT, computed tomography; SARS-CoV-2, severe acute respiratory syndrome coronavirus type 2; USG, ultrasonography

Physicians most frequently agreed with the statement that the benefits of a CT scan in septic patients outweigh possible side effects such as radiation exposure or risks of contrast media administration (90.5%, *n* = 336/371) (Fig. 2). Consensus was found regardless of work experience, workplace, and medical specialty—with radiologists agreeing the most (S1–S3). Avoidance of a CT scan in patients with sepsis due to radiation exposure was widely rejected by participating physicians (Fig. 2). Physicians with < 3 years of work experience tended to reject the statement less strongly than those with more seniority (S1). Most participants (87.0%, *n* = 323/371) opposed conducting an unenhanced CT scan to avoid adverse effects of ICM administration in septic patients (Fig. 2)—radiologists were the most likely to disapprove (Tables 1, S2, S3).

### Indications for contrast media administration

**Fig. 3:**
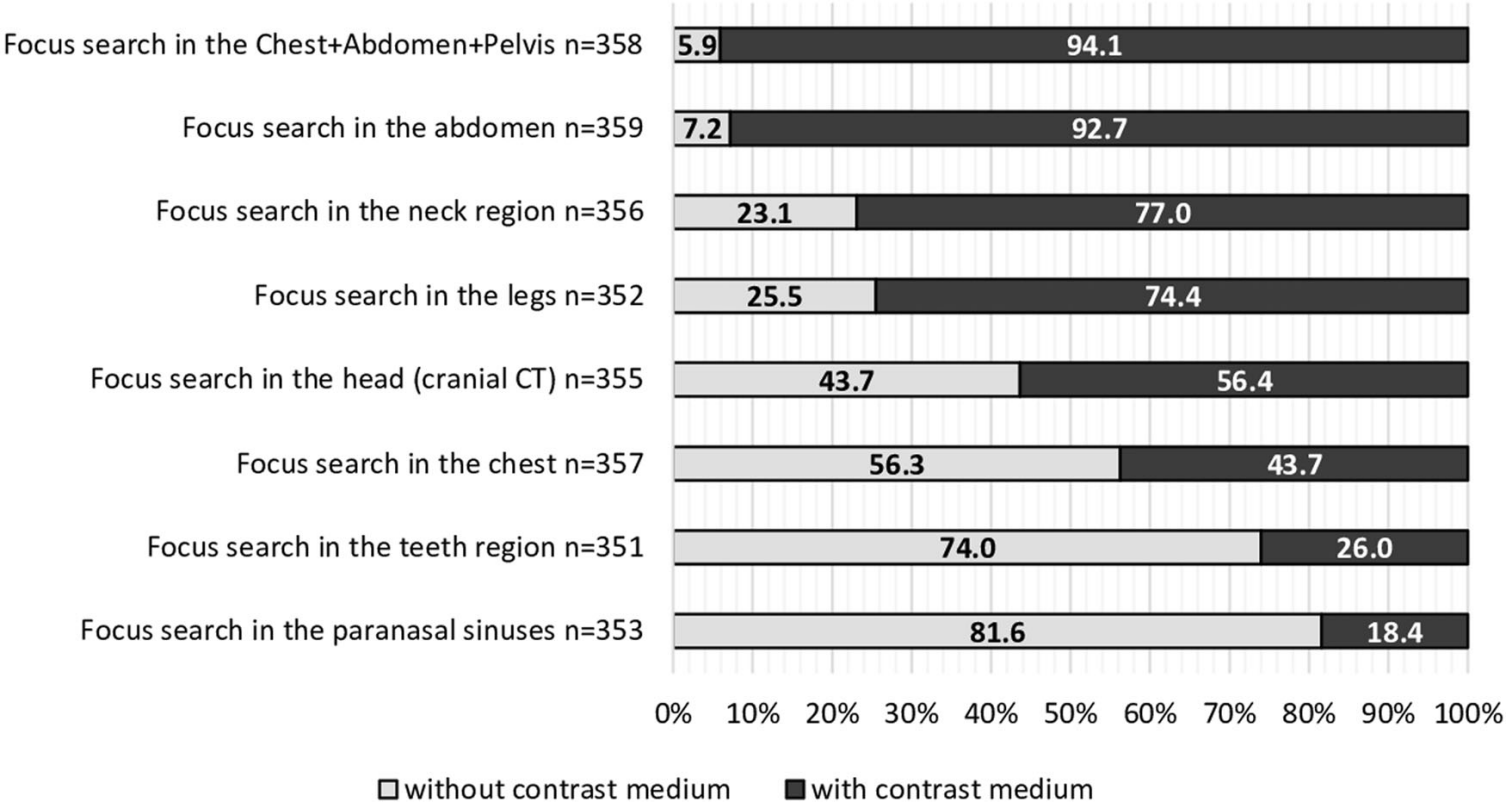
Perspectives on the usefulness of contrast medium in focus detection dependent on body region. Administration of ICM in patients with sepsis for focus search in the abdomen by CT was widely agreed upon (92.7%, *n* = 333/359). For focus search in the chest region, 56.3% (*n* = 201/357) would request an unenhanced CT. 94.1% (*n* = 337/358) of physicians favored a CECT when examining the chest, abdomen, and pelvis together: while 54.2% (*n* = 194/358) would conduct the CT examination definitely with ICM, 39.9% (*n* = 143/358) favored a CT scan preferably with ICM. Physicians chose to request a CT with ICM in scans of the neck (77.0%, *n* = 274/356) and the legs (74.4%, *n* = 262/352). 43.7% (*n* = 155/355) of participants chose to conduct a cranial CT without ICM, while 56.4% (*n* = 200/355) favored an examination with ICM. The body regions for which participants most strongly opposed CECT were the teeth (74.0%, *n* = 260/351) and paranasal sinuses (81.6%, *n* = 288/353). CT, computed tomography; ICM, iodinated contrast media; CECT, contrast-enhanced CT. Without contrast medium = answers “definitely without” and “preferably without” counted together; with contrast medium = answers “definitely with” and “preferably with” counted together

**Fig. 4:**
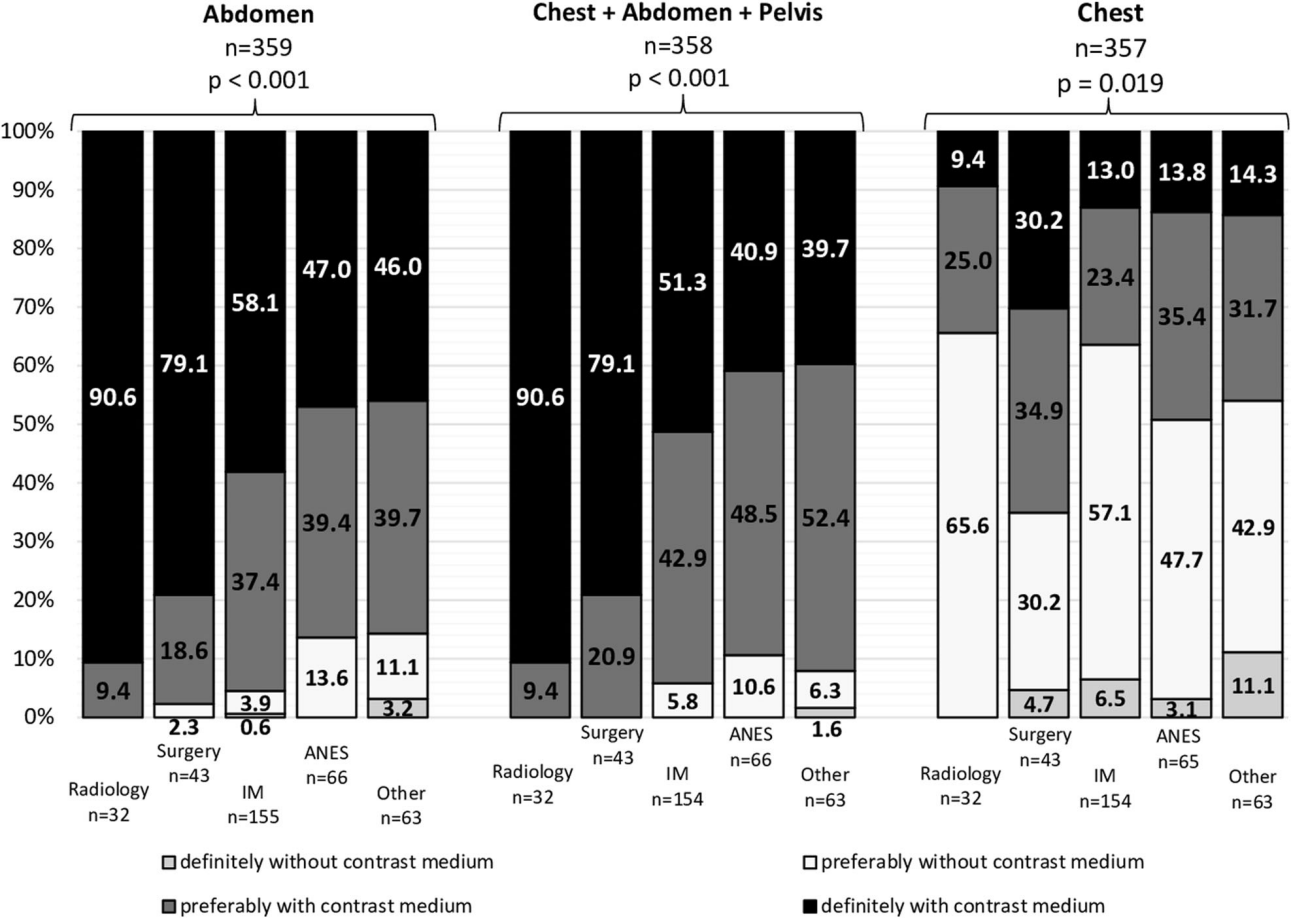
Influence of medical specialty on the perspective on the indication of contrast medium with regard to the body region examined. Radiologists (90.6%, *n* = 29/32) and surgeons (79.1%, *n* = 34/43) saw definite indications for contrast medium administration for both a CT of the abdomen and for a whole-body CT in septic patients. Meanwhile, for both CT examination options, the groups internal medicine, anesthesiology, and other were torn between a definite or preferable CECT. The percentage of physicians from these three specialties opting for a preferable CECT scan was notably higher than that of radiologists or surgeons. Anesthesiologists were the group most likely to rather choose an unenhanced whole-body CT scan (10.6%, *n* = 7/66). Only one physician selected “definitely without contrast medium” administration for a whole-body CT (1.6%, *n* = 1/6). While 65.6% (*n* = 21/32) of radiologists and 63.6% (*n* = 98/154) of internal medicine physicians preferred an unenhanced CT scan for focus search in the chest region, 65.1% (*n* = 28/43) of surgeons saw a preferable or definite indication for a CECT examination. Overall, radiologists not once selected the option “definitely without contrast medium” for any of the three options. IM, internal medicine; ANES, anesthesiology; CT, computed tomography; whole-body CT, combined CT examination of the chest, abdomen, and pelvis; CECT, contrast-enhanced CT. No bar = 0.0% (*n* = 0)

Physicians saw the strongest indication for ICM administration in septic patients for focus search with (1) a CT of the abdomen and (2) a whole-body CT (Fig. 3). Participants with a work experience of > 11 to ≤ 20 years were more likely to approve ICM administration when conducting a focal search by CT of the abdomen (98.4%, *n* = 60/61) and even more likely when performing a whole-body CT (100.0%, *n* = 60/60) (Table 1). More physicians from radiology than from other departments considered a CT of the abdomen (90.3%, *n* = 28/31) and a whole-body CT (90.3%, *n* = 28/31) as valid indications for ICM administration (Table 1). A clear preference was not evident regarding CT examinations performed to look for chest infections. Here, 56.3% (*n* = 201/357) of all participants preferred an unenhanced CT examination, whereas 43.7% (*n* = 156/357) would request CECT. A closer look at the statistically significant differences in responses among physicians from different medical specialties revealed that radiologists’ and surgeons’ desire for contrast administration was striking for both a CT of the abdomen and a whole-body CT in septic patients (Fig. 4). Other specialties were more likely to opt for preferable rather than definite performance of a CECT. Additionally, surgeons more frequently saw a preferable or definite indication for a CECT of the chest than any other specialties. Of note, radiologists did not once select a definite indication for an unenhanced CT in any of the three CT examination options (Fig. 4). While, overall, the request of a CECT was strongly supported for examinations of the neck and legs, unenhanced scans were preferred for examining teeth and paranasal sinuses (Fig. 3).

### Perspectives on contraindications to conduct a CECT

**Fig. 5:**
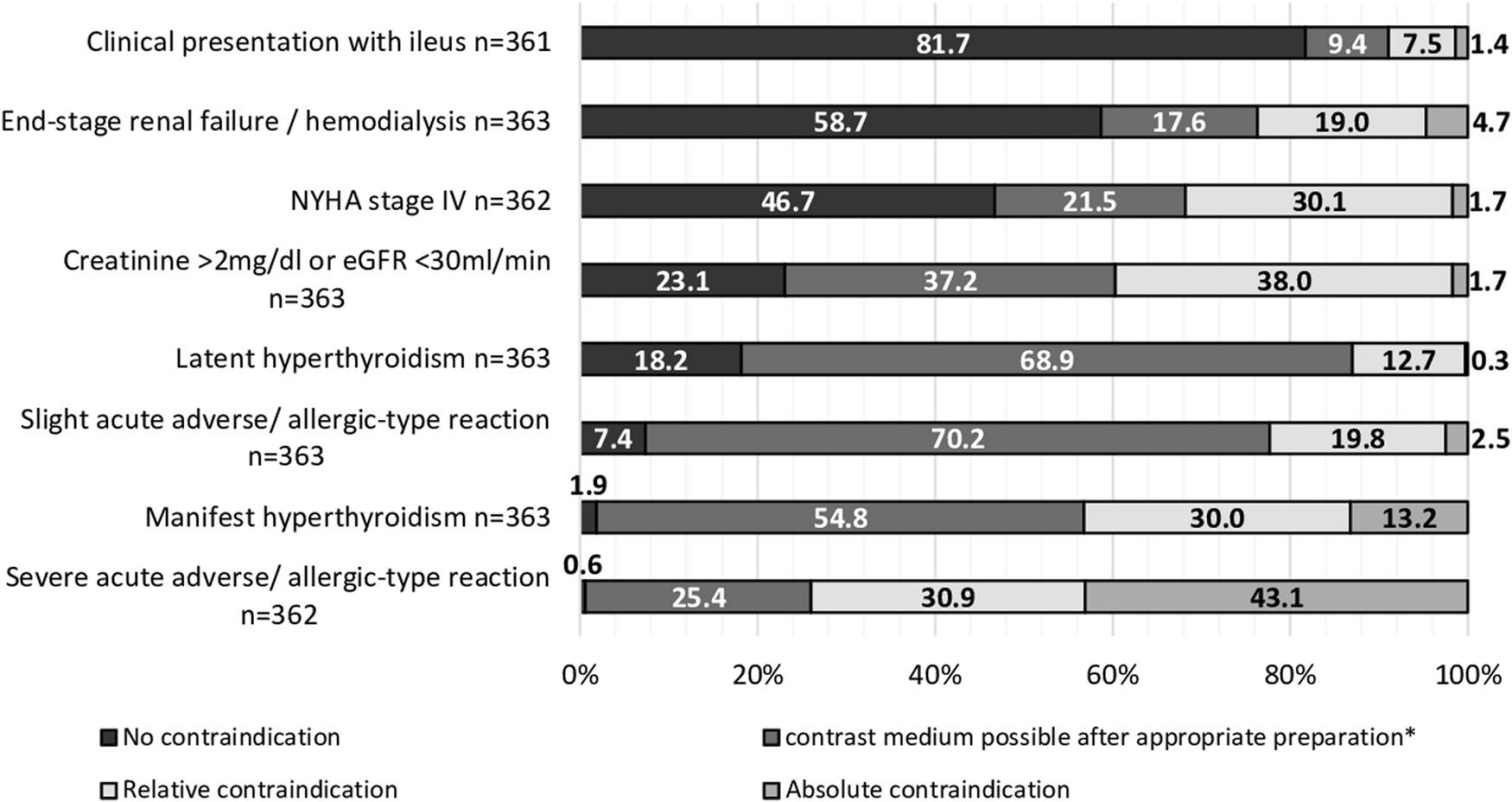
Perspectives on contraindications to the use of contrast medium in septic patients. Physicians regarded a severe acute adverse reaction to an administered ICM in the past as the most important absolute contraindication to ICM use in septic patients (43.1%, *n* = 156/362). Conversely, a septic patient’s clinical presentation with an ileus was most frequently considered no contraindication to CECT (81.7%, *n* = 295/361), followed by end-stage renal failure or need for hemodialysis (58.7%, *n* = 213/363) and NYHA stage IV symptoms (46.7%, *n* = 169/362) in a septic patient. Overall, physicians saw mostly no contraindication to administering ICM to a septic patient with NYHA stage IV symptoms. The option of ICM administration after adequate preparation was mostly chosen for a slight acute adverse reaction in the past (70.2%, *n* = 255/363) and a patient with latent hyperthyroidism (68.9%, *n* = 250/363). 54.8% (*n* = 199/363) of physicians also indicated that administration of ICM would be possible after preparation in septic patients with manifest hyperthyroidism. Only 0.3% (*n* = 1/363) of physicians chose latent hyperthyroidism as an absolute contraindication, compared to 13.2% (*n* = 48/363) for manifest hyperthyroidism. And, conversely, only 1.9% (*n* = 7/363) of participants saw no contraindication in the manifest form, whereas it was 18.2% (*n* = 66/363) for latent hyperthyroidism. Most likely to be chosen as relative contraindications to ICM administration in septic patients were creatinine values > 2 mg/dL or an eGFR < 30 mL/min (38.0%, *n* = 138/363). More detailed information can be found in Supplementary Figs. S4–S6. CT, computed tomography; CECT, contrast-enhanced CT; ICM, iodinated contrast media; TSH, thyroid-stimulating hormone; fT3, free triiodothyronine; eGFR, estimated glomerular filtration rate; NYHA, New York Heart Association. Manifest hyperthyroidism = TSH reduced, fT3 increased; latent hyperthyroidism = TSH reduced, normal fT3; *preparation = prophylaxis (including hydration and/or medication) or lower dose of contrast medium

Overall, participants rarely considered any of the listed conditions as an absolute contraindication to ICM administration in patients with sepsis – except for a history of severe acute adverse reaction after previous ICM administration (Fig. 5). A slight allergic reaction after previously administered ICM was the condition most frequently considered to require preparation before a CECT examination (70.2%, *n* = 255/363). Again, there were differences in responses among medical specialties (Tables 1, S4). Regardless of their specialty, however, physicians expressed concern about a CECT examination in patients with a history of a severe allergic reaction after earlier ICM administration (S4). For septic patients with a creatinine level > 2 mg/dL or an estimated glomerular filtration rate (eGFR) < 30 mL/min needing a CT scan to locate an infectious focus, responses ranged from approving ICM administration after adequate patient preparation to viewing it as a relative contraindication (Fig. 5). Only 6.3% (*n* = 2/32) of radiologists but 23.1% (*n* = 84/363) of all participants did not consider a CECT to be contraindicated in such cases (S5). The clinical presentation of end-stage renal failure or a renal replacement indication in a septic patient was not seen as a contraindication for conducting a CECT by 58.7% (*n* = 213/363) of all participants (Fig. 5). While radiologists not once indicated this condition as an absolute contraindication for a CECT, one-fifth of the “other medical specialty” group did (S5). For patients with either latent (68.9%, *n* = 250/363) or manifest (54.8%, *n* = 199/363) hyperthyroidism, the most common choice was appropriate preparation before ICM administration. Latent hyperthyroidism was rarely seen as an absolute contraindication, while manifest hyperthyroidism was more frequently considered one. Radiologists stood out as the group most frequently considering manifest hyperthyroidism an absolute contraindication to CECT in septic patients, whereas other specialties rarely chose this option (Tables 1, S6).

### Perceptions on the procedure after a focus-negative initial CT examination

**Fig. 6:**
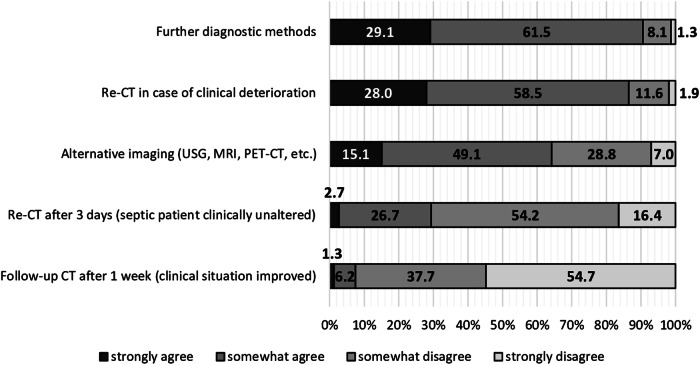
Perspectives on further management if CT fails to detect an infectious focus. Our survey revealed that, after an initial CT failed to identify a source of infection, physicians would rely on further diagnostic testing—with a total of 61.5% ticking the response option “somewhat agree” (*n* = 228/371) and 29.1% “strongly agree” (*n* = 108/371). The second most common option selected was to repeat CT in patients with clinical deterioration (86.5%, *n* = 321/371). More than half of the participants (64.2%, *n* = 238/371) reported resorting to alternative imaging modalities. When the septic patient’s clinical situation improved, the vast majority of physicians declined to request a follow-up CT 1 week after initial CT—with 54.7% (*n* = 203/371) strongly and 37.7% (*n* = 140/371) somewhat disagreeing. 70.6% (*n* = 262/371) of participants would not order a repeat CT after 3 days if the patient was clinically unaltered. Further details on descriptive data can be found in the supplementary material (Tables S7–S9). re-CT, repeat computed tomography; USG, ultrasonography; MRI, magnetic resonance imaging; PET-CT, positron emission tomography and computed tomography; CT, computed tomography

The most agreed upon procedure for focus identification, if an initial CT failed to detect a source of infection in a septic patient, was to rely on further diagnostic tests (Fig. 6). There was consensus regardless of physicians’ work experience and medical specialty (S7, S9). However, compared to other workplaces, ICU physicians’ responses gravitated significantly to rely on further diagnostic test (S8). Most participants (64.2%, *n* = 238/371) had a fairly positive attitude toward using alternative imaging modalities after a focus-negative initial CT scan (Fig. 6). Here, radiologists were most likely to strongly disagree (S8, S9). Performing a repeat CT scan (re-CT) in septic patients with clinical deterioration was considered a good option in case of an initial focus-negative CT (Fig. 6). In clinically unchanged septic patients, most participants stated they would prefer not to request a re-CT after 3 days. Again, radiologists were the medical specialty most frequently (27.3%, *n* = 9/33) strongly disagreeing (S9). However, physicians from the operating room (OR) and outpatient clinic were most likely to somewhat agree with a re-CT 3 days after an initial scan when the patient’s condition was clinically unchanged (S8).

## Discussion

### Summary

There is agreement among the participants of our questionnaire survey that the benefits of a CT scan in septic patients are greater than the potential adverse effects of radiation exposure or ICM administration. They see the strongest indication for a CECT when searching for an infectious focus in the abdomen alone or simultaneously in the abdomen, chest, and pelvis regions. A history of a severe allergic reaction after contrast administration was considered a relative to absolute contraindication. However, in patients with a history of mild adverse effects or manifest hyperthyroidism, respondents generally favored the option of specific preparation prior to CECT. If an initial CT scan cannot detect a source of infection, doctors recognize alternative and/or accompanying diagnostic tools as an essential part of managing sepsis. At the same time, the participants perceived repeated CT scans as a good option for septic patients with clinical deterioration.

### Literature

To our knowledge, this is the first study to explore physicians’ perspectives on the use of CT in patients with sepsis, with no other literature available on this topic aside from our previous work [18]. Despite exposure to ionizing radiation, imaging remains a crucial component of clinical practice for diagnosing various conditions [8, 13, 15]. Advances in technology and adherence to the ALARA (as low as reasonably achievable) principle have constantly reduced the risks associated with ionizing radiation over recent decades [13, 25, 26]. Nevertheless, any CT examination must be justified, meaning the benefits should outweigh the risks [14, 27]. The sepsis guidelines emphasize the rapid search for foci of infection but do not explicitly state that sepsis justifies a CT indication [5, 6]. The participants of our survey regard the prompt identification of an infection source in septic patients as a justified indication for a CT scan and the associated radiation exposure. However, participants’ caution is evident in their reluctance to perform repeat CT scans after 3 days in clinically unaltered or follow-up scans after 1 week in clinically improved septic patients, demonstrating their intent to minimize unnecessary radiation exposure.

The use of contrast media is necessary for certain CT examinations and has been shown to improve diagnostic accuracy [14, 28]. In guidelines for the general use of CT, CECT is explicitly recommended for the assessment of abdominal and soft tissue infections [12, 14, 28, 29]. Consistent with these guideline recommendations, our participants identified the strongest indication for a CECT when examining the abdomen or a combined examination of the chest, abdomen, and pelvis. In our survey, there was agreement between experienced physicians and radiologists that infectious foci in the abdominal cavity are challenging to detect on unenhanced CT scans.

Before administering ICM, the patient’s individual risk of adverse reactions should be assessed [29–31]. Given that the literature describes mild adverse reactions as typically self-limiting [19, 31], participants considered the use of CECT with premedication to be appropriate. Still, the majority of physicians (43.1%) considered a history of severe adverse reactions to ICM as an absolute contraindication for CECT in septic patients. Interestingly, we have shown before that 75% of final-year medical students judge this as an absolute contraindication [24]. Similarly, Alchallah et al found that 65.4% of medical students saw a severe allergic reaction as an absolute contraindication [32]. Fortunately, the risk of moderate to severe adverse events has decreased in recent decades (incidence < 0.04%), largely due to the development of safer ICMs [19, 30, 31]. Nonetheless, we must be aware that severe adverse reactions can cause life-threatening conditions [19, 31].

Another concern in clinical practice is the development of acute renal failure due to the administration of ICM, known as CIN. In accordance with the literature, our participants considered ICM administration rather safe in patients with end-stage renal failure requiring dialysis [19]. However, in less progressed chronic kidney disease (CKD) with residual kidney function, a higher risk of developing CIN has been discussed [19, 20, 33]. Consistent with this, physicians expressed concern about ICM use in septic patients with a creatinine level > 2 mg/dL or eGFR < 30 mL/min. Currently, premedication and other prophylactic measures, such as appropriate hydration, are suggested for patients with CKD and an eGFR < 30 mL/min [19, 20, 29, 31, 34]. The incidence of CIN ranges from around 2% in the general population to around 30% in patients at a high risk of developing kidney disease [19, 35]. Davenport et al argue that the risk of AKI associated with ICM administration has been overstated [34]. Accordingly, Tong et al reported no statistically significant increase in the risk of CIN among patients undergoing CECT compared to those examined by unenhanced CT [36]. The findings of Hsu et al show no increased risk of AKI in septic patients undergoing CECT, highlighting AKI as a potential complication of sepsis [37].

Physicians participating in our survey were aware of the risks associated with ICM administration in patients with hyperthyroidism. The European Society of Emergency Radiology and the European Thyroid Association list hyperthyroidism as a contraindication to the administration of ICM [31, 38]. However, in emergency situations where CECT is necessary, Bednarczuk et al state that even patients with manifest hyperthyroidism can undergo the scan with premedication as a preventive measure [38]. Consistent with this, most participants favored premedication over a uniform rejection of CECT in septic patients with hyperthyroidism. While there is no literature on the preferred choices of different medical specialties, our participating radiologists stood out as being more skeptical of using ICM in patients with manifest hyperthyroidism compared to other specialties.

Overall, most published studies and guidelines on the use of CT or contrast media do not specifically address septic patients. The question that remains is whether sepsis should be considered an extreme emergency, possibly warranting less focus on the risks and adverse effects of CT and ICM in clinical decision-making, or whether potential adverse events pose an even greater risk to septic patients.

### Limitations

Even though only a minority of physicians surveyed responded, i.e., approximately 15%, this response rate can be deemed sufficient for providing an overview of physicians’ perspectives on the survey’s topic. Our center comprises three university hospitals, offering a diverse and substantial patient population as well as varied perspectives from healthcare professionals. This setup allowed us to generate robust data and meaningful insights within a single-center framework. While our data reflect the viewpoints of physicians from different settings involved in the management of sepsis, these findings may not accurately represent physicians’ perspectives from different healthcare facilities or different countries. However, our results reflect the heterogeneous variety of work experiences, workplaces, and physicians’ medical specialties in managing septic patients. Lastly, our survey used closed-ended questions with a limited number of options instead of open-ended questions. Therefore, we decided to use 4-point scales instead of 5-point scales to at least avoid the limitation of participants’ tendency to choose the middle of the response spectrum on a Likert scale.

### Conclusion

Physicians across different specialties, workplaces, and work experience levels view CT as a diagnostic pillar for managing patients with sepsis. However, detailed research is needed to assess the risks associated with contrast media and the diagnostic value of CECT in identifying infectious foci in septic patients. Future studies should aim at generating better evidence on the use of CT in septic patients to achieve the best outcome.

## Supplementary information

ELECTRONIC SUPPLEMENTARY MATERIAL

## Data Availability

We state that complete data will not be shared. Due to strict local data protection laws, we will not provide all readers of the manuscript access to the raw data in a publicly accessible repository. We do not wish to publish the questionnaire as it is still being used for other unpublished studies.

## Abbreviations

AKI: Acute kidney injury
CECT: Contrast-enhanced computed tomography
CIN: Contrast-induced nephropathy
CKD: Chronic kidney disease
CT: Computed tomography
eGFR: Estimated glomerular filtration rate
fT3: Free triiodothyronine
ICM: Iodinated contrast media
MRI: Magnetic resonance imaging
NYHA: New York Heart Association
PET-CT: Positron emission tomography and computed tomography
re-CT: Repeat computed tomography
TSH: Thyroid-stimulating hormone
USG: Ultrasonography
Whole-body CT: Combined CT examination of the chest, abdomen, and pelvis
