# Supplementary material for: Critical reflection on the indication for computed tomography: an interdisciplinary survey of risk and benefit management in patients with sepsis
Source: Insights Imaging. 2025 Jan 13;16:15. doi: 10.1186/s13244-024-01894-3 (PMC11730041; doi:10.1186/s13244-024-01894-3)
Supplement: Supplementary file 1 — ELECTRONIC SUPPLEMENTARY MATERIAL [file 13244_2024_1894_MOESM1_ESM.pdf]

Critical reflection on the indication for computed tomography: An interdisciplinary survey of risk and benefit management in patients with sepsis
ELECTRONIC SUPPLEMENTARY MATERIAL

		Work experience in years (n=371)																			
		<3 years n=74				>3-≤7 years n=130				>7-≤11 years n=73				>11-≤20 years n=64				>20 years n=30			
		1	2	3	4	1	2	3	4	1	2	3	4	1	2	3	4	1	2	3	4
Clinical scenarios - how to manage patients with sepsis	I perform a CT because the benefits outweigh the side effects	2.7 (2)	8.1 (6)	44.6 (33)	44.6 (33)	2.3 (3)	3.8 (5)	44.6 (58)	49.2 (64)	2.7 (2)	9.6 (7)	41.1 (30)	46.6 (34)	1.6 (1)	7.8 (5)	39.1 (25)	51.6 (33)	6.7 (2)	6.7 (2)	46.7 (14)	40.0 (12)
	In patients with a confirmed SARS-CoV-2 infection, I definitely perform a CT scan	25.7 (19)	37.8 (28)	24.3 (18)	12.2 (9)	23.8 (31)	33.8 (44)	32.3 (42)	10.0 (13)	23.3 (17)	39.7 (29)	27.4 (20)	9.6 (7)	14.1 (9)	32.8 (21)	37.5 (24)	15.6 (10)	23.3 (7)	23.3 (7)	33.3 (10)	20.0 (6)
	Before performing a CT, there should be an USG examination. The subsequent CT serves to find additional or new foci	20.3 (15)	33.8 (25)	35.1 (26)	10.8 (8)	20.0 (26)	43.8 (57)	28.5 (37)	7.7 (10)	23.3 (17)	35.6 (26)	38.4 (28)	2.7 (2)	15.6 (10)	46.9 (30)	28.1 (18)	9.4 (6)	26.7 (8)	23.3 (7)	33.3 (10)	16.7 (5)
	Before performing a CT, there should be an USG examination. The subsequent CT serves to confirm the findings	18.9 (14)	40.5 (30)	35.1 (26)	5.4 (4)	22.3 (29)	52.3 (68)	19.2 (25)	6.2 (8)	32.9 (24)	39.7 (29)	23.3 (17)	4.1 (3)	25.0 (16)	48.4 (31)	25.0 (16)	1.6 (1)	26.7 (8)	43.3 (13)	16.7 (5)	13.3 (4)
	Due to radiation exposure, I avoid CT whenever possible	48.6 (36)	41.9 (31)	8.1 (6)	1.4 (1)	60.8 (79)	31.5 (41)	6.2 (8)	1.5 (2)	63.0 (46)	30.1 (22)	6.8 (5)	0.0 (0)	62.5 (40)	31.3 (20)	6.3 (4)	0.0 (0)	70.0 (21)	16.7 (5)	3.3 (1)	10.0 (3)
	I prefer to conduct unenhanced CT scans to avoid adverse effects of contrast media administration.	41.9 (31)	36.5 (27)	18.9 (14)	2.7 (2)	53.1 (69)	36.2 (47)	9.2 (12)	1.5 (2)	47.9 (35)	42.5 (31)	9.6 (7)	0.0 (0)	62.5 (40)	25.0 (16)	12.5 (8)	0.0 (0)	60.0 (18)	30.0 (9)	6.7 (2)	3.3 (1)

Table S1 Influence of work experience on CT indications in sepsis (absolute and relative frequencies)

At 35.1% (n=26/74), participants with less than three years of work experience were most likely to "somewhat agree" that a USG should be performed first, with a CT scan used to confirm findings in septic patients. Overall, physicians most frequently somewhat disagreed with this statement. Physicians from all work experience disagreed that a USG examination should be performed before a CT scan, which in turn serves to find new or additional foci. Physicians with over 20 years of experience were most likely to "strongly agree" (16.7%, n=5/30). Physicians with more than three years of experience generally rejected avoiding CT scans due to radiation concerns, while those with less than three years more often "somewhat disagreed" (41.9%, n=31/74). Regardless of experience, participants did not support unenhanced CT scans due to concerns about contrast media.

1= strongly disagree; 2= somewhat disagree; 3= somewhat agree; 4= strongly agree
CT= computed tomography; SARS-CoV-2 = Severe acute respiratory syndrome coronavirus type 2; USG= ultrasonography

		Workplace (n=371)																											
		ICU n=115				General ward n=82				Emergency department n=50				OR n=42				Radiology department n=32				Outpatient clinic n=32				Other n=18			
		1	2	3	4	1	2	3	4	1	2	3	4	1	2	3	4	1	2	3	4	1	2	3	4	1	2	3	4
Clinical scenarios - how to manage patients with sepsis	I perform a CT because the benefits outweigh the side effects	0.9 (1)	4.3 (5)	36.5 (42)	58.3 (67)	2.4 (2)	8.5 (7)	48.8 (40)	40.2 (33)	8.0 (4)	6.0 (3)	44.0 (22)	42.0 (21)	2.4 (1)	11.9 (5)	42.9 (18)	42.9 (18)	0.0 (0)	3.1 (1)	34.4 (11)	62.5 820)	3.1 (1)	9.4 (3)	59.4 (19)	28.1 (9)	5.6 (1)	5.6 (1)	44.4 (8)	44.4 (8)
	In patients with a confirmed SARS-CoV-2 infection, I definitely perform a CT scan	19.1 (22)	28.7 (33)	30.4 (35)	21.7 (25)	32.9 (27)	34.1 (28)	29.3 (24)	3.7 (3)	28.0 (14)	40.0 (20)	26.0 (13)	6.0 (3)	14.3 (6)	38.1 (16)	35.7 (15)	11.9 (5)	28.1 (9)	40.6 (13)	21.9 (7)	9.4 (3)	3.1 (1)	37.5 (12)	50.0 (16)	9.4 (3)	22.2 (4)	38.9 (7)	22.2 (4)	16.7 (3)
	Before performing a CT, there should be an USG examination. The subsequent CT serves to find additional or new foci	22.6 (26)	42.6 (49)	27.8 (32)	7.0 (8)	18.3 (15)	47.6 (39)	26.8 (22)	7.3 (6)	12.0 (6)	28.0 (14)	44.0 (22)	16.0 (8)	21.4 (9)	40.5 (17)	35.7 (15)	2.4 (1)	40.6 (13)	34.3 (11)	15.6 (5)	9.4 (3)	9.4 (3)	37.5 (12)	46.9 (15)	6.3 (2)	22.2 (4)	16.7 (3)	44.4 (8)	16.7 (3)
	Before performing a CT, there should be an USG examination. The subsequent CT serves to confirm the findings	32.3 (37)	44.3 (51)	18.3 (21)	5.2 (6)	23.2 (19)	54.9 (45)	17.1 (14)	4.9 (4)	8.0 (4)	48.0 (24)	34.0 (17)	10.0 (5)	16.7 (7)	45.2 (19)	35.7 (15)	2.4 (1)	50.0 (16)	37.5 (12)	12.5 (4)	0.0 (0)	18.8 (6)	37.5 (12)	37.5 (12)	6.3 (2)	11.1 (2)	44.4 (8)	33.3 (6)	11.1 (2)
	Due to radiation exposure, I avoid CT whenever possible	59.1 (68)	32.2 (37)	8.7 (10)	0.0 (0)	48.8 (40)	41.5 (34)	6.1 (5)	3.7 (3)	62.0 (31)	32.0 (16)	6.0 (3)	0.0 (0)	61.9 (26)	33.3 (14)	4.8 (2)	0.0 (0)	68.8 (22)	21.9 (7)	6.3 82)	3.1 (1)	71.9 (23)	18.8 (6)	6.3 (2)	3.1 (1)	66.7 (12)	27.8 (5)	0.0 (0)	5.6 (1)
	I prefer to conduct unenhanced CT scans because I am concerned about contrast media side effects	52.2 (60)	33.9 (39)	13.0 (15)	0.9 (1)	42.7 (35)	39.0 (32)	15.9 (13)	2.4 (2)	46.0 (23)	42.0 (21)	12.0 (6)	0.0 (0)	54.8 (23)	31.0 (13)	11.9 (5)	2.4 (1)	93.8 (30)	6.3 (2)	0.0 (0)	0.0 (0)	40.6 (13)	50.0 (16)	9.4 (3)	0.0 (0)	50.0 (9)	38.9 (7)	5.6 (1)	5.6 (1)

Table S2 Influence of workplace on CT indications in different scenarios (absolute and relative frequencies)

The radiology department was the only group that not once strongly agreed that a USG examination should be performed before a CT examination, which in turn is used to confirm the USG findings. Moreover, they were the group that strongly disagreed most often (50.0%, n=16/32). In contrast, emergency department

physicians were least likely to strongly disagree (8.0%, n=4/50) with that statement. Instead, the emergency physicians argued that a USG examination should be performed before a CT scan, which in turn serves to find new or additional foci (60.0%, n=30/50). Meanwhile, 75.0% (n=24/32) of physicians from the radiology department strongly or somewhat disagree with that clinical scenario. A heterogeneous response distribution can be observed for the statement to perform a CT scan on patients with a confirmed SARS-CoV-2 infection. Compared to the other groups, the intensive care physicians stood out. They were the most likely to answer "strongly agree" (21.7%, n=25/115). However, only 3.7% (n=3/82) of physicians from the general ward chose that option. Radiology department physicians responded similarly: only 9.4% (n=3/32) strongly agreed, while 28.1% (n=9/32) strongly disagreed on a definite CT examination in SARS-CoV-2 confirmed patients. Compared to other workplaces, more physicians from the radiology department (93.8%, n=30/32) strongly opposed performing an unenhanced CT scan because of possible contrast media side effects.

1= strongly disagree; 2= somewhat disagree; 3= somewhat agree; 4= strongly agree

CT= computed tomography; SARS-CoV-2 = Severe acute respiratory syndrome coronavirus type 2; USG= ultrasonography; ICU= Intensive care unit; OR = Operating room

		Medical specialty (n=370)																			
		Internal medicine n=157				Surgery n=44				Radiology n=33				Anesthesiology n=70				Other n=66			
		1	2	3	4	1	2	3	4	1	2	3	4	1	2	3	4	1	2	3	4
Clinical scenarios - how to manage patients with sepsis	I perform a CT because the benefits outweigh the side effects	4.5 (7)	5.1 (8)	45.9 (72)	44.6 (70)	0.0 (0)	9.1 (4)	22.7 (10)	68.2 (30)	0.0 (0)	3.0 81)	36.4 (12)	60.6 (20)	2.9 (2)	7.1 (5)	51.4 (36)	38.6 (27)	1.5 (1)	10.6 (7)	45.5 (30)	42.4 (28)
	In patients with a confirmed SARS-CoV-2 infection, I definitely perform a CT scan	22.3 (35)	32.5 (51)	31.2 (49)	14.0 (22)	29.5 (13)	34.1 (15)	29.5 (13)	6.8 (3)	27.3 (9)	42.4 (14)	21.2 (7)	9.1 (3)	17.1 (12)	32.9 (23)	34.3 (24)	15.7 (11)	21.2 (14)	39.4 (26)	30.3 (20)	9.1 (6)
	Before performing a CT, there should be an USG examination. The subsequent CT serves to find additional or new foci	14.0 (22)	38.2 (60)	38.2 (60)	9.6 (15)	38.6 (17)	45.5 (20)	11.4 (5)	4.5 (2)	39.4 (13)	36.4 (12)	15.2 (5)	9.1 (3)	15.7 (11)	45.7 (32)	34.3 (24)	4.3 (3)	19.7 (13)	30.3 (20)	37.9 (25)	12.1 (8)
	Before performing a CT, there should be an USG examination. The subsequent CT serves to confirm the findings	18.5 (29)	50.3 (79)	24.8 (39)	6.4 (10)	31.8 (14)	54.4 (24)	9.1 (4)	4.5 (2)	48.5 (16)	39.4 (13)	12.1 (4)	0.0 (0)	20.0 (14)	50.0 (35)	28.6 (20)	1.4 (1)	27.3 (18)	28.8 (19)	33.3 (22)	10.6 (7)
	Due to radiation exposure, I avoid CT whenever possible	55.4 (87)	38.2 (60)	5.1 (8)	1.3 (2)	79.5 (35)	9.1 (4)	11.4 (5)	0.0 (0)	69.7 (23)	21.2 (7)	6.1 (2)	3.0 (1)	54.3 (38)	34.3 (24)	11.4 (8)	0.0 (0)	57.6 (38)	36.4 (24)	1.5 (1)	4.5 (3)
	I prefer to conduct unenhanced CT scans because I am concerned about contrast media side effects	45.9 (72)	40.1 (63)	12.7 (20)	1.3 (2)	77.3 (34)	18.2 (8)	4.5 (2)	0.0 (0)	90.9 (30)	9.1 (3)	0.0 (0)	0.0 (0)	34.3 (24)	48.6 (34)	14.3 (10)	2.9 (2)	48.5 (32)	33.3 (22)	16.7 (11)	1.5 (1)

Table S3 Influence of medical specialty on CT indications in different scenarios (absolute and relative frequencies)

A similar heterogenous percentage distribution of responses from the different specialties was observed in the clinical scenario of performing a CT scan in patients with a confirmed SARS-CoV-2 infection. However, radiologists were least likely to agree (30.3%, n=10/33). Compared to the other medical specialties, radiologists more frequently strongly disagreed (48.5%, n=16/33) that a USG examination should be performed before a CT scan, which in turn is used to confirm USG findings. Radiologists (39.4 %, n=13/33) and surgeons (38.6 %, n=17/44) were most likely to reject the statement that a USG examination should be performed before a CT scan, which in turn serves to find new or additional foci. Radiologists' rejection of the statement was emphasized by no one choosing the "strongly agree" answer. The response spectrum of the rest of the medical specialties was distributed across the response options "somewhat agree" and "strongly agree." Especially radiologists (90.9%, n=30/33) and surgeons (77.3%, n=34/44) strongly disapproved of conducting an unenhanced CT examination due to concerns about contrast media side effects. Avoiding a CT scan in septic patients due to radiation exposure was widely rejected. Above all, surgeons strongly disagreed with that clinical scenario (79.5%, n=35/44).

1= strongly disagree; 2= somewhat disagree; 3= somewhat agree; 4= strongly agree

CT= computed tomography; SARS-CoV-2 = Severe acute respiratory syndrome coronavirus type 2; USG= ultrasonography

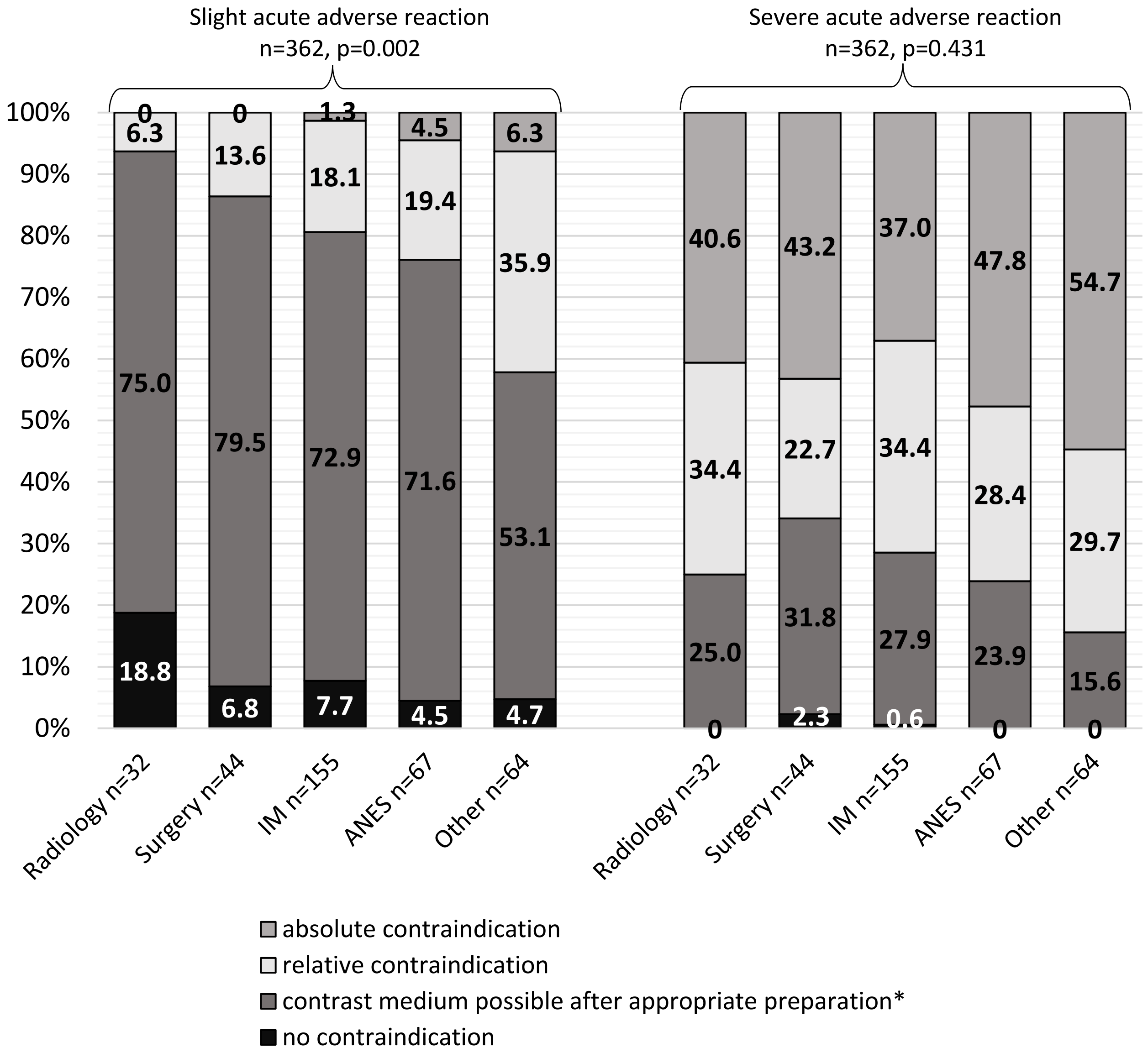

Figure S4 Perspectives on contraindications for contrast media application in a septic patient with a history of adverse reactions after CECT

Internal medicine, surgery, anesthesiology, and radiology specialists frequently chose the need for appropriate preparation before contrast media application in a patient with a history of a slight adverse reaction to contrast media. At the same time, the "other medical specialty" group was more likely to view it as a relative contraindication (35.9%, n=23/64). Additionally, primarily radiologists saw no contraindication for a CECT (18.8%, n=6/32).

In septic patients with a history of a severe adverse reaction to contrast medias, the least favorite answer was "no contraindication." With no significant differences between the specialties, physicians showed concern about using contrast medias in these cases. All specialty groups favored the answer "absolute contraindication."

*IM = Internal medicine; ANES = Anesthesiology; Other = Other specialties; *preparation= prophylaxis (including hydration and/or medication) or lower dose of contrast media; CECT = Contrast-enhanced computed tomography*

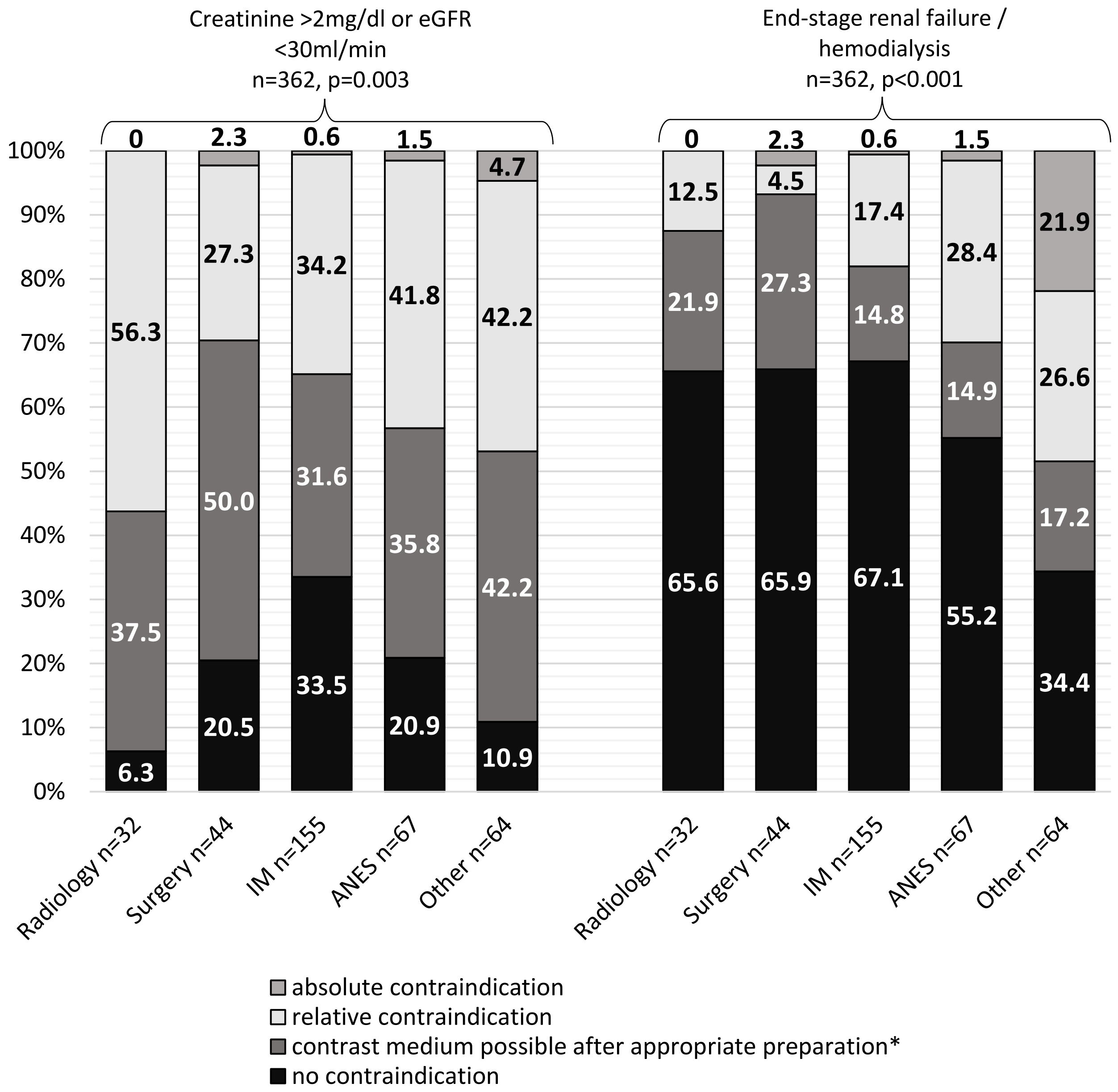

Figure S5 Perspectives on contraindication for contrast media application in a septic patient with signs of kidney failure

For a focus search in a septic patient with creatinine levels >2mg/dl or an estimated glomerular filtration rate (eGFR) <30ml/min, the responses were equally distributed between the need of preparation and relative contraindication. However, only 6.3% (n=2/32) of radiologists but 33.5% (n=52/155) of internal medicine physicians saw no contraindication for a CECT.
Aside from the group "other," end-stage renal failure or the need for dialysis in a septic patient was stated as no contraindication by the majority of the specialties. While radiologists not once saw an absolute contraindication for a CECT in a septic patient with end-stage renal failure, 21.9% (n=14/64) of the "other medical specialty" group did. At 28.4% (n=19/67), anesthesiologists were the group most likely to choose a relative contraindication.

*IM = Internal medicine; ANES = Anesthesiology; Other = Other specialties; *preparation= prophylaxis (including hydration and/or medication) or lower dose of contrast media; CECT = Contrast-enhanced computed tomography*

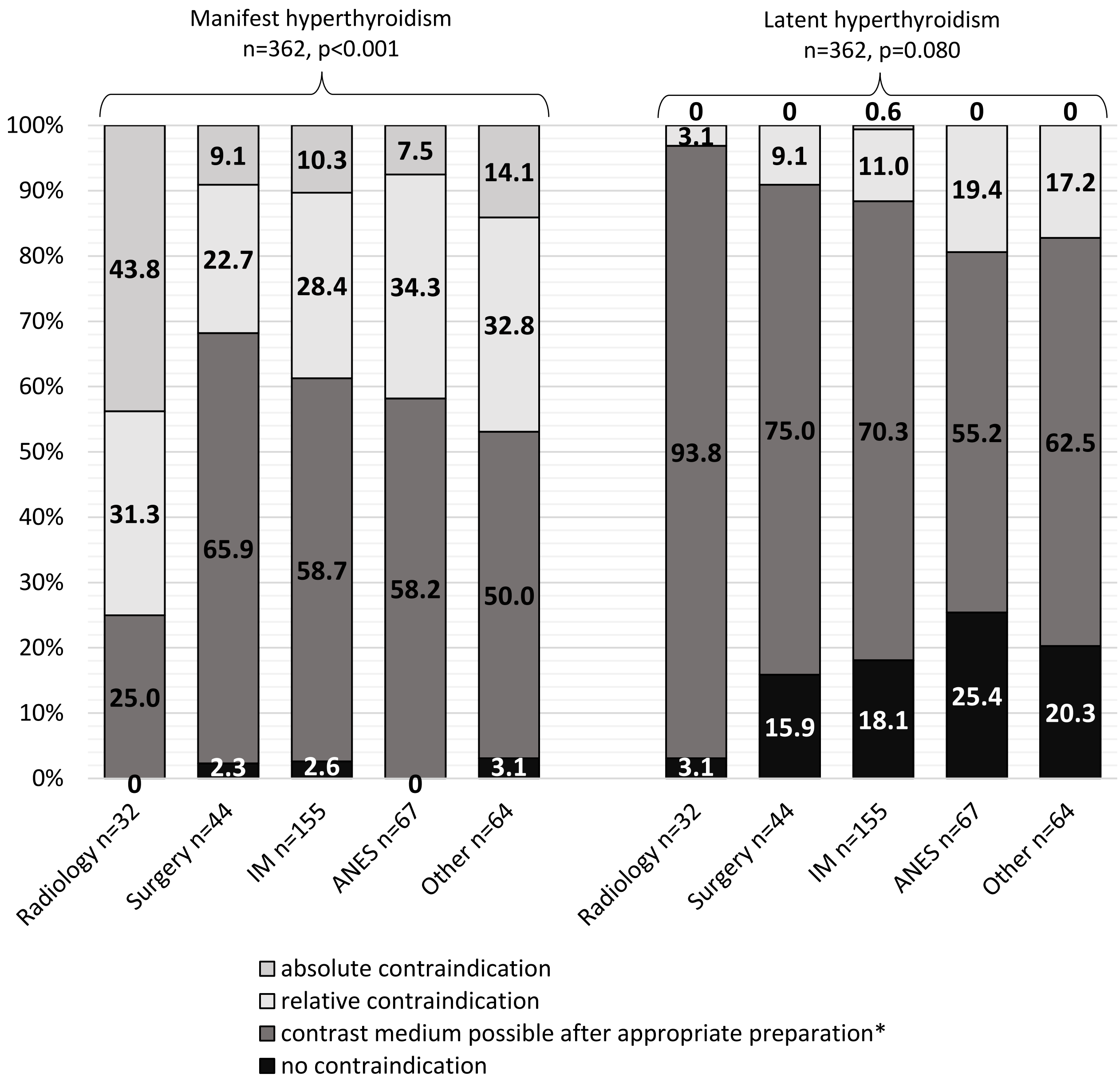

<u>Figure S6</u> <u>Perspectives on contraindication for contrast media application in a septic patient with hyperthyroidism</u>

At 43.8% (n=14/32), radiologists indicated an absolute contraindication for CECT in septic patients with manifest hyperthyroidism. Meanwhile, surgeons (65.9 %, n=29/44), internists (58.7 %, n=91/155), and anesthesiologists (58.2 %, n=39/67) all considered appropriate preparation required before performing CECT. In contrast, only a quarter of radiologists chose this option.

Radiologists stood out, with 93.8% (n=30/32) of them choosing the need for preparation before administering contrast medias in a septic patient with latent hyperthyroidism. The other groups also most frequently chose that response. However, the alternative answers were selected by a higher proportion than the radiologists. For example, 25.4% (n=17/67) of anesthesiologists and only 3.1% (n=1/32) of radiologists saw no contraindication for a CECT in a patient with sepsis and latent hyperthyroidism. Similarly, only 3.1% (n=1/32) of radiologists but 11.0% (n=17/155) of physicians from internal medicine responded with a relative contraindication.

*IM = Internal medicine; ANES = Anesthesiology; Other= Other specialties; *preparation= prophylaxis (including hydration and/or medication) or lower dose of contrast media;*

CECT = Contrast-enhanced computed tomography

		**Work experience in years (n=371)**																			
		<3 years n=74				>3-≤7 years n=130				>7-≤11 years n=73				>11-≤20 years n=64				>20 years n=30			
		1	2	3	4	1	2	3	4	1	2	3	4	1	2	3	4	1	2	3	4
**how to proceed if no source of infection was detected with an initial CT scan**	Relying on further diagnostic methods	1.4 (1)	5.4 (4)	64.9 (48)	28.4 (21)	0.8 (1)	7.7 (10)	59.2 (77)	32.3 (42)	1.4 (1)	6.8 (5)	63.0 (46)	28.8 (21)	3.1 (2)	9.4 (6)	62.5 (40)	25.0 (16)	0.0 (0)	16.7 (5)	56.7 (17)	26.7 (8)
	Re-CT in case of clinical deterioration	0.0 (0)	10.8 (8)	56.8 (42)	32.4 (24)	2.3 (3)	12.3 (16)	63.8 (83)	21.5 (28)	1.4 (1)	12.3 (9)	63.0 (46)	23.3 (17)	0.0 (0)	9.4 (6)	53.1 (34)	37.5 (24)	10.0 (3)	13.3 (4)	40.0 (12)	36.7 (11)
	Alternative imaging (USG, MRI, PET-CT, etc.)	5.4 (4)	32.4 (24)	44.6 (33)	17.6 (13)	10.0 (13)	30.0 (39)	47.7 (62)	12.3 (16)	6.8 (5)	27.4 (20)	54.8 (40)	11.0 (8)	4.7 (3)	31.3 (20)	51.6 (33)	12.5 (8)	3.3 (1)	13.3 (4)	46.7 (14)	36.7 (11)
	Re-CT after 3 days (patient clinically unaltered)	14.9 (11)	52.7 (39)	32.4 (24)	0.0 (0)	16.9 (22)	52.3 (68)	27.7 (36)	3.1 (4)	20.5 (15)	57.5 (42)	21.9 (16)	0.0 (0)	10.9 (7)	60.9 (39)	23.4 (15)	4.7 (3)	20.0 (6)	43.3 (13)	26.7 (8)	10.0 (3)
	Follow-up CT after 1 week (clinical situation improved)	45.9 (34)	45.9 (34)	6.8 (5)	1.4 (1)	56.9 (74)	34.6 (45)	8.5 (11)	0.0 (0)	53.4 (39)	41.1 (30)	4.1 (3)	1.4 (1)	59.4 (38)	34.4 (22)	4.7 (3)	1.6 (1)	60.0 (18)	30.0 (9)	3.3 (1)	6.7 (2)

Table S7 Influence of work experience on the perception of the procedure after a focus-negative initial CT examination (absolute and relative frequencies)

A preference for further diagnostic methods after a focus-negative CT was detectable regardless of the physicians' work experience. However, the more professional experience a physician had, the more likely they disagreed with this preference. For instance, 16.7% (n=5/30) of physicians with >20 years of experience chose the answer "somewhat disagree." The group of physicians with >20 years of experience was also most opposed to performing a re-CT in septic patients with clinical deterioration (23.3%, n=7/30). At 78.0% (n=57/73), physicians with >7-≤11 years of experience firmly refused to perform a re-CT in a clinically unaltered septic patient. Furthermore, a follow-up CT one week after an initial focus-negative CT in a clinically improved patient was overwhelmingly rejected by physicians of all experience levels, with more than 90% choosing either "strongly disagree" or "somewhat disagree." Responses on the use of alternative imaging modalities were quite heterogeneous. The group with >20 years of work experience stands out, as 36.7% (n=11/30) strongly favor using alternative imaging methods.

1= strongly disagree; 2= somewhat disagree; 3= somewhat agree; 4= strongly agree

re-CT= repeat computed tomography; USG= ultrasonography; MRI= magnetic resonance imaging; PET-CT= positron emission tomography and computed tomography; CT= computed tomography

how to proceed if no source of infection was detected with an initial CT scan	Workplace (n=371): ICU n=115				General ward n=82				Emergency department n=50				OR n=42				Radiology department n=32				Outpatient clinic n=32				Other n=18			
	1	2	3	4	1	2	3	4	1	2	3	4	1	2	3	4	1	2	3	4	1	2	3	4	1	2	3	4
Relying on further diagnostic methods	1.7 (2)	4.3 (5)	53.9 (62)	40.0 (46)	0.0 (0)	6.1 85)	63.4 (52)	30.5 (25)	4.0 (2)	8.0 (4)	64.0 (32)	24.0 (12)	2.4 (1)	7.1 (3)	69.0 (29)	21.4 (9)	0.0 (0)	15.6 (5)	68.8 (22)	15.6 (5)	0.0 (0)	25.0 (8)	62.5 (29)	12.5 (4)	0.0 (0)	0.0 (0)	61.1 (11)	38.9 (7)
Re-CT in case of clinical deterioration	0.9 (1)	17.4 (20)	50.4 (58)	31.3 (36)	1.2 (1)	8.5 (7)	64.6 (53)	25.6 (21)	0.0 (0)	12.0 (6)	60.0 (30)	28.0 (14)	2.4 (1)	14.3 (6)	52.4 (22)	31.0 (13)	3.1 (1)	3.1 (1)	59.4 (19)	34.4 (11)	3.1 (1)	6.3 (2)	68.8 (22)	21.9 (7)	11.1 (2)	5.6 (1)	72.2 (13)	11.1 (2)
Alternative imaging (USG, MRI, PET-CT, etc.)	3.5 (4)	30.4 (35)	51.3 (59)	14.8 (17)	9.8 (8)	32.9 (27)	45.1 (37)	12.2 (10)	6.0 (3)	18.0 (9)	64.0 (32)	12.0 (6)	9.5 (4)	33.3 (14)	50.0 (21)	7.1 (3)	21.9 (7)	28.1 (9)	37.5 (12)	12.5 (4)	0.0 (0)	25.0 (8)	40.6 (13)	34.4 (11)	0.0 (0)	27.8 (5)	44.4 (8)	27.8 (5)
Re-CT after 3 days (patient clinically unaltered)	16.5 (19)	60.9 (70)	18.3 (21)	4.3 (5)	11.0 (9)	57.3 (47)	29.3 (24)	2.4 (2)	16.0 (8)	62.0 (31)	18.0 (9)	4.0 (2)	16.7 (7)	40.5 (17)	40.5 (17)	2.4 (1)	28.1 (9)	31.3 (10)	40.6 (13)	0.0 (0)	12.5 (4)	46.9 (15)	40.6 (13)	0.0 (0)	27.8 (5)	61.1 (11)	11.1 (2)	0.0 (0)
Follow-up CT after 1 week (clinical situation improved)	53.0 (61)	36.5 (42)	8.7 (10)	1.7 (2)	51.2 (42)	41.5 (34)	6.1 (5)	1.2 (1)	72.0 (36)	28.0 (14)	0.0 (0)	0.0 (0)	52.4 (22)	42.9 (18)	4.8 (2)	0.0 (0)	65.6 (21)	28.1 (9)	6.3 (2)	0.0 (0)	43.8 (14)	43.8 (14)	6.3 (2)	6.3 (2)	38.9 (7)	50.0 (9)	11.1 (2)	0.0 (0)

Table S8 Influence of workplace on the perception of the procedure after a focus-negative initial CT examination (absolute and relative frequencies)

With 40.0% (n=46/115), ICU physicians tended to strongly agree that further diagnostic methods after a CT examination could not detect a source of infection. On the contrary, physicians from the radiology department (15.6%, n=5/32) and outpatient clinic (25.0%, n=8/32) were the most likely to rather disagree in this regard. Participants from all workplaces most frequently chose to "somewhat agree" on using alternative imaging modalities. Especially physicians from ICU (67.9%, n=76/115), emergency departments (76.0%, n=38/50), and outpatient clinics (75.0%, n=24/32) reported alternative imaging modalities as good options. Participants from the radiology department were most likely to disagree strongly (21.9%, n=7%32). Performing a re-CT in septic patients with clinical deterioration was widely supported. In contrast, conducting a follow-up CT examination when the clinical situation had improved was vehemently opposed, with emergency department physicians unanimously rejecting it. Requesting a re-CT in a clinically unaltered septic patient three days after an initial focus-negative CT was generally rather refused. However, some physicians from specific workplaces expressed a desire to perform a re-CT in such cases. For example, 42.9% (n=18/42) from the OR and 40.6% (n=13/32) from the radiology department somewhat or strongly agreed.

1= strongly disagree; 2= somewhat disagree; 3= somewhat agree; 4= strongly agree

re-CT= repeat computed tomography; USG= ultrasonography; MRI= magnetic resonance imaging; PET-CT= positron emission tomography and computed tomography; CT= computed tomography; ICU= Intensive care unit; OR = Operating room

		Medical specialty (n=370)																			
		Internal medicine n=157				Surgery n=44				Radiology n=33				Anesthesiology n=70				Other n=66			
		1	2	3	4	1	2	3	4	1	2	3	4	1	2	3	4	1	2	3	4
how to proceed if no source of infection was detected with an initial CT	Relying on further diagnostic methods	1.3 (2)	6.4 (10)	62.4 (98)	29.9 (47)	0.0 (0)	9.1 (4)	65.9 (29)	25.0 (11)	0.0 (0)	15.2 (5)	69.7 (23)	15.2 (5)	2.9 (2)	5.7 (4)	60.0 (42)	31.4 (22)	1.5 (1)	10.6 (7)	53.0 (35)	34.8 (23)
	Re-CT in case of clinical deterioration	1.9 (3)	12.1 (19)	63.1 (99)	22.9 (36)	2.3 (1)	9.1 (4)	54.5 (24)	34.1 (15)	3.0 (1)	3.0 (1)	60.6 (20)	33.3 (11)	1.4 (1)	15.7 (11)	50.0 (35)	32.9 (23)	1.5 (1)	12.1 (8)	59.1 (39)	27.3 (18)
	Alternative imaging (USG, MRI, PET-CT, etc.)	4.5 (7)	25.5 (40)	53.5 (84)	16.6 (26)	11.4 (5)	47.7 (21)	34.1 (15)	6.8 (3)	21.2 (7)	27.3 (9)	39.4 (13)	12.1 (4)	4.3 (3)	32.9 (23)	50.0 (35)	12.9 (9)	6.1 (4)	19.7 (13)	53.0 (35)	21.2 (14)
	Re-CT after 3 days (patient clinically unaltered)	15.9 (25)	63.7 (100)	15.3 (24)	5.1 (8)	9.1 (4)	38.6 (17)	50.0 (22)	2.3 (1)	27.3 (9)	33.3 (11)	39.4 (13)	0.0 (0)	20.0 (14)	54.3 (38)	25.7 (18)	0.0 (0)	13.6 (9)	53.0 (35)	31.8 (21)	1.5 (1)
	Follow-up CT after 1 week (clinical situation improved)	58.0 (91)	31.8 (50)	7.0 (11)	3.2 (5)	50.0 (22)	45.5 (20)	4.5 (2)	0.0 (0)	63.6 (21)	30.3 (10)	6.1 (2)	0.0 (0)	51.4 (36)	44.3 (31)	4.3 (3)	0.0 (0)	50.0 (33)	42.4 (28)	7.6 (5)	0.0 (0)

Table S9 Influence of medical specialty on the perception of the procedure after a focus-negative initial CT examination (absolute and relative frequencies)

A preference for relying on further diagnostic methods was detectable regardless of the physicians' medical specialty. With 15.2% (n=5/32) ticking the answer option "somewhat disagree," radiologists showed the lowest percentage of agreement. 70.1% (n=110/157) of internists and 74.2% (n=49/66) of physicians with other specialties stated to conduct alternative imaging methods to search for a focus after a focus-negative CT scan. Here, radiologists had the highest proportion of strongly disagreeing participants (21.2%, n=7/33). Additionally, 59.1% (n=26/44) of surgeons showed disagreement. A follow-up CT in a patient with clinical improvement after one week was widely opposed. On the contrary, physicians showed great interest in a re-CT in septic patients with clinical deterioration. There were differences in the responses regarding an indication for a re-CT in a clinically unaltered septic patient. Radiologists were most likely to strongly disagree (27.3%, n=9/33) with performing a re-CT three days after a focus-negative CT in a clinically unaltered septic patient. In contrast, surgeons most commonly agreed somewhat (50.0%, n=22/44). The rest favored the answer "somewhat disagree."

1= strongly disagree; 2= somewhat disagree; 3= somewhat agree; 4= strongly agree

re-CT= repeat computed tomography; USG= ultrasonography; MRI= magnetic resonance imaging; PET-CT= positron emission tomography and computed tomography; CT= computed tomography
